# Epigenomics in an extraterrestrial environment: organ-specific alteration of DNA methylation and gene expression elicited by spaceflight in *Arabidopsis thaliana*

**DOI:** 10.1186/s12864-019-5554-z

**Published:** 2019-03-12

**Authors:** Mingqi Zhou, Natasha J. Sng, Collin E. LeFrois, Anna-Lisa Paul, Robert J. Ferl

**Affiliations:** 1 Plant Molecular and Cellular Biology Program, University of Florida, Gainesville, FL USA; 2 Horticultural Sciences Department, University of Florida, Gainesville, FL USA; 3 Interdisciplinary Center for Biotechnology, University of Florida, Gainesville, FL USA

**Keywords:** Spaceflight, Microgravity, Arabidopsis, Transcriptome, DNA methylation, Epigenome, Epigenetic, Veggie, ISS

## Abstract

**Background:**

Plants adapted to diverse environments on Earth throughout their evolutionary history, and developed mechanisms to thrive in a variety of terrestrial habitats. When plants are grown in the novel environment of spaceflight aboard the International Space Station (ISS), an environment completely outside their evolutionary history, they respond with unique alterations to their gene expression profile. Identifying the genes important for physiological adaptation to spaceflight and dissecting the biological processes and pathways engaged by plants during spaceflight has helped reveal spaceflight adaptation, and has furthered understanding of terrestrial growth processes. However, the underlying regulatory mechanisms responsible for these changes in gene expression patterns are just beginning to be explored. Epigenetic modifications, such as DNA methylation at position five in cytosine, has been shown to play a role in the physiological adaptation to adverse terrestrial environments, and may play a role in spaceflight as well.

**Results:**

Whole Genome Bisulfite Sequencing of DNA of Arabidopsis grown on the ISS from seed revealed organ-specific patterns of differential methylation compared to ground controls. The overall levels of methylation in CG, CHG, and CHH contexts were similar between flight and ground DNA, however, thousands of specifically differentially methylated cytosines were discovered, and there were clear organ-specific differences in methylation patterns. Spaceflight leaves had higher methylation levels in CHG and CHH contexts within protein-coding genes in spaceflight; about a fifth of the leaf genes were also differentially regulated in spaceflight, almost half of which were associated with reactive oxygen signaling.

**Conclusions:**

The physiological adaptation of plants to spaceflight is likely nuanced by epigenomic modification. This is the first examination of differential genomic methylation from plants grown completely in the spaceflight environment of the ISS in plant growth hardware developed for informing exploration life support strategies. Yet even in this optimized plant habitat, plants respond as if stressed. These data suggest that gene expression associated with physiological adaptation to spaceflight is regulated in part by methylation strategies similar to those engaged with familiar terrestrial stress responses. The differential methylation maps generated here provide a useful reference for elucidating the layers of regulation of spaceflight responses.

**Electronic supplementary material:**

The online version of this article (10.1186/s12864-019-5554-z) contains supplementary material, which is available to authorized users.

## Background

Plants physiologically adapt to spaceflight using strategies that mimic several aspects of familiar terrestrial environmental responses, even though the spaceflight response is distinct from any singular terrestrial response. Among those strategies are transcriptomic and proteomic alterations that are increasingly well defined as more experiments are conducted on the International Space Station (ISS). Plants have a long history in spaceflight research (recent reviews include: [1, 2]). Because of the relationship between gravity and plant architecture [3], plants are considered important tools for discovery of gravity-related biological phenomena [4]. In addition, plant growth in support of closed ecology human life support systems places plant space biology in both discovery and applications roles in the space exploration science agenda.

Near typical plant growth can occur in space within the advanced growth hardware of the ISS. However, evidence from multiple experiments, using a variety of plant species, plant tissues, and in a variety of spaceflight habitats, strongly suggests that physiological adaptation to spaceflight requires an altered state of metabolism and structure that is conditioned by differential gene expression. Early spaceflight gene expression studies suggested that modifications in calcium signaling were important to the metabolic adaptation of plants to spaceflight [5–9] but did not suggest any singular or particular environmental stress that characterized spaceflight. More recent work on the International Space Station has enabled several highly replicated transcriptome experiments that reveal more about the global transcriptional response of *Arabidopsis thaliana* (Arabidopsis), and data now exists for several different cultivars, cell types and growth habitats [6, 8, 10–23]. Of particular note is that there are substantial differences in gene expression patterns even among tissue sources of the same genotype; Arabidopsis leaves, hypocotyls, roots, and root tips all have markedly different spaceflight transcriptomes, as do undifferentiated cell cultures [13, 14, 16, 18].

Many genes differentially expressed during spaceflight are also differentially expressed during familiar, yet distinct terrestrial stress responses, including stresses involving pathogens, drought, temperature and salt. There are several categories of genes that are well-represented in a variety of terrestrial stress responses that are also well-represented in the spaceflight response. Genes associated with cell wall remodeling, Reactive Oxygen Species signaling, light signal transduction, hormone metabolism, and calcium signaling are represented in multiple abiotic and biotic stress responses on Earth (e.g. [24–36]. The spaceflight environment appears to engage similar cross talk among these genes, and transcriptomic and proteomic evidence can be found from a variety of orbital experiments comprising a diversity of plant systems (e.g. [14–16, 18, 20–23, 37–46]. The abundance of representative genes from these categories across cultivars, missions and even species suggests that spaceflight is perceived by plants as an environment requiring a physiological response for acclimation.

Terrestrial plant stresses are increasingly understood to include significant epigenetic changes in the DNA methylome, and epigenome changes have been shown to influence the adaptability of the next generation to a particular stress. Current thought is that a dynamic epigenome plays a much larger role in plant adaptation that previously envisioned. Recent work in plant communities challenged with recurrent stressful events suggests that the epigenetic variation of the plants in these ecosystems are part of the adaptive strategies [47], and epigenetic regulation has been implicated in the acquisition of herbicide resistance in plant populations [48]. Epigenetic responses have also been well documented as important to strategies for pathogen resistance (reviewed in [49, 50]) and in coping with abiotic stressors such as salt, cold, and drought [51–57] and at least some of these epigenomic alterations appear to be transmitted through seeds. Further, elucidation of the nature of those epigenome changes can inform genetic modification strategies to breed plants that are more resistant or adaptable to that stress. The same strategy can be applied to any stressor that elicits an epigenomic response, potentially including spaceflight.

Many of the genes that are differentially expressed by plants in spaceflight intersect with gene networks associated with pathogen attack, herbivory, salt stress and drought [14–16, 18–23, 44]. These stresses are well-documented elicitors of differential DNA methylation [49, 51–54, 58–66]. Therefore the epigenome plays a substantial role in the physiological adaptation to terrestrial biotic [50, 58, 60–62], and abiotic stress [57, 67–71]. The determination of Arabidopsis differential methylation in response to spaceflight would provide insight into the role the epigenome plays in the physiological adaptation to spaceflight. This study examined the role of DNA methylation, in particular, 5mCyt, in spaceflight-grown plants with the expectation of elucidating functional linkages between epigenetics and spaceflight adaptation.

In plants, DNA methylation occurs in the CG, CHG and CHH context (where H is A, C, or T), each of which is maintained through cellular divisions by a distinct pathway and set of enzymes including DDM1, VIMs and MET1 (CG), SUVH4 deposited H3K9me2 and CMT3 (CHG), and CMT2 (CHH) [72–74].

Also of note is that the unique environment of spaceflight also comes with unique challenges for experimental design and data. Standard protocols and approaches typical for determining the genome-wide methylome were modified to accommodate limitations in preservation techniques and limited sample sizes of spaceflight-grown plants. This study therefore utilized Arabidopsis plants preserved on orbit in RNAlater™ for transcriptomics studies, in order to make the first determination of spaceflight-induced changes in the Arabidopsis DNA methylome.

## Results

### Arabidopsis seedlings demonstrated near typical terrestrial growth patterns in the veggie hardware of ISS

**Fig. 1:**
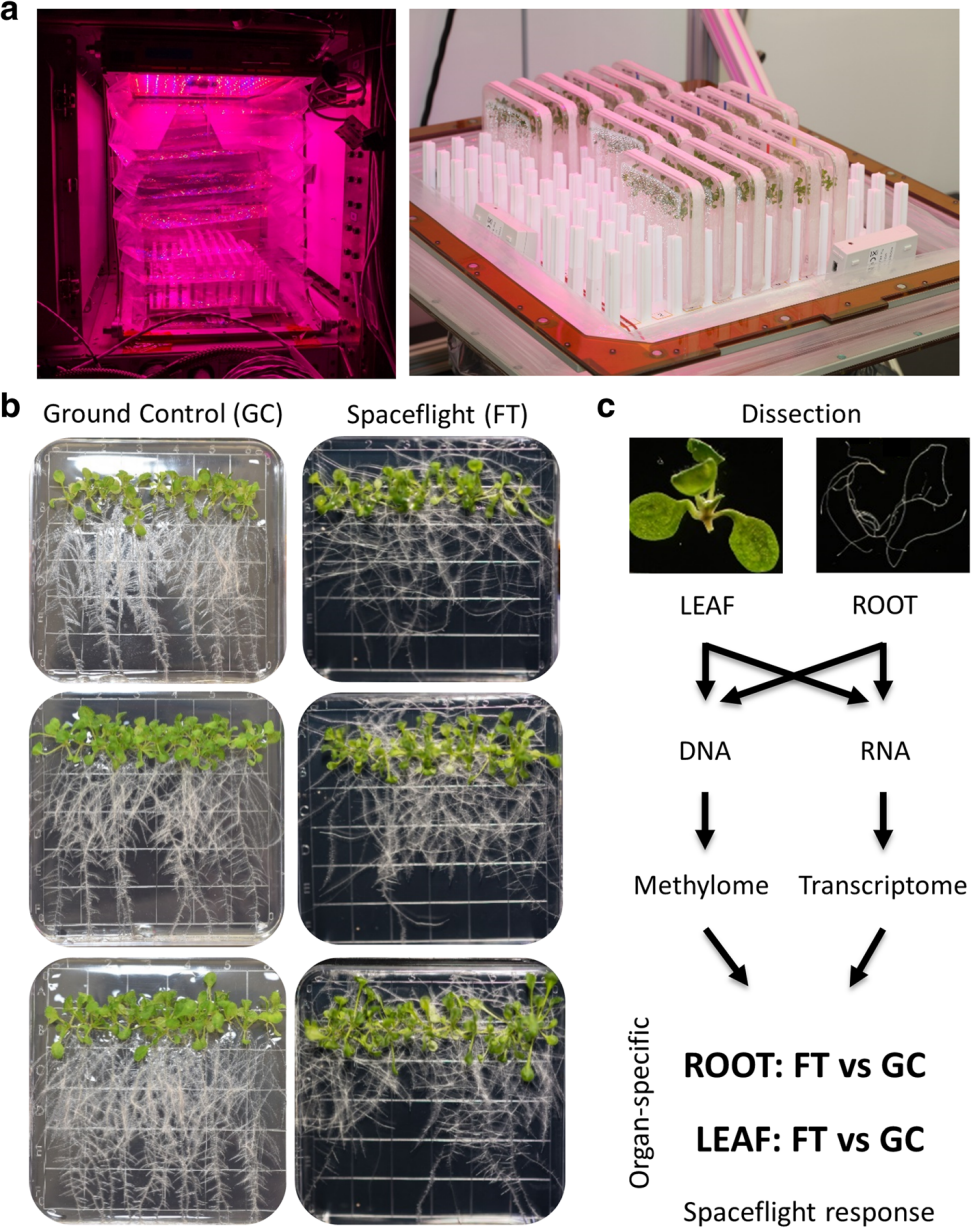
The phenotype of Arabidopsis seedlings grown on the ISS and on the ground. **a** A view of the APEX03–2 experiment growing inside Veggie on the ISS (left) and a view of plates installed in the ground Veggie unit to illustrate the configuration of plates installed vertically in racks. **b** *Arabidopsis thaliana* (WS) seedlings were grown under constant LED light conditions in the Vegetable Production System (VPS/Veggie) hardware on the Columbus Module of the ISS for 11 days. Corresponding ground control plants were grown in the Veggie hardware within the ISS Environmental Simulation (ISSES) chamber at Kennedy Space Center for 11 days. Each plate was considered a biological replicate and a total of 6 plates were used in this experiment. All three biological replicates for both spaceflight and ground control are represented. **c** The workflow of transcriptome and DNA methylome analysis. Plant materials harvested in RNAlater were dissected into root and leaf tissues. Total RNA & DNA were isolated separately, and the differential analyses were conducted in an organ-specific manner comparing the effects of spaceflight (FT) vs ground control (GC) in the roots and leaves

The three plates of Arabidopsis plants showed near typical germination and plant growth within the Veggie hardware of the ISS. Figure 1a provides a view of the APEX03–2 experiment growing inside Veggie on orbit (left) alongside a view of plates installed in the ground unit at Kennedy Space Center. Figure 1b shows the actual plates from the ISS (Spaceflight FT) and the related Ground Control plates. General growth and morphology on orbit was similar to that on the ground, with roots growing on the surface of the medium and typical development of shoots and leaves. Without gravity as a directional cue, the roots of the spaceflight plants grew in a generally negatively phototropic manner, but as can be seen in the right-hand column of Fig. 1b, the directional growth of the roots is not uniform in spaceflight.

Compared to the ISS growth systems with strong directional light like the Advanced Biological Research System (ABRS) [75], the lighting system of Veggie was more intense, and yet more diffuse with respect to directionality (Fig. 1a). However, WS seedlings grown on the ISS in Veggie showed sufficient negative phototropism of roots, and positive phototropism of shoots relative to the LED light source to substantially mimic the growth patterns of the vertically-grown ground controls in the duplicate Veggie hardware housed in the KSC ISSES chamber (Fig. 1b).

For molecular analyses, the seedlings were dissected into leaves and roots by removing the hypocotyl (Fig. 1c top). Leaf and root samples from each of the three plates shown in Fig. 1a were subjected to RNA-sequencing (RNA-seq) for transcriptome analysis and Whole Genome Bisulfite Sequencing (WGBS) for methylome analysis (Fig. 1c), providing three biological replicates for each treatment/organ combination.

### Spaceflight did not induce large scale changes in DNA methylation, but the distribution of methylation levels was slightly modified by spaceflight

WGBS revealed the genome-wide DNA methylation patterns of spaceflight-grown plants along with their comparable ground controls. As a check on the conversion process, the correctly bisulfite converted and mapped cytosine sites in chloroplast DNA of all samples were found to be near 99% (Additional file 1: Table S1), indicating a good rate of conversion and processing in the experimental procedures.

**Fig. 2:**
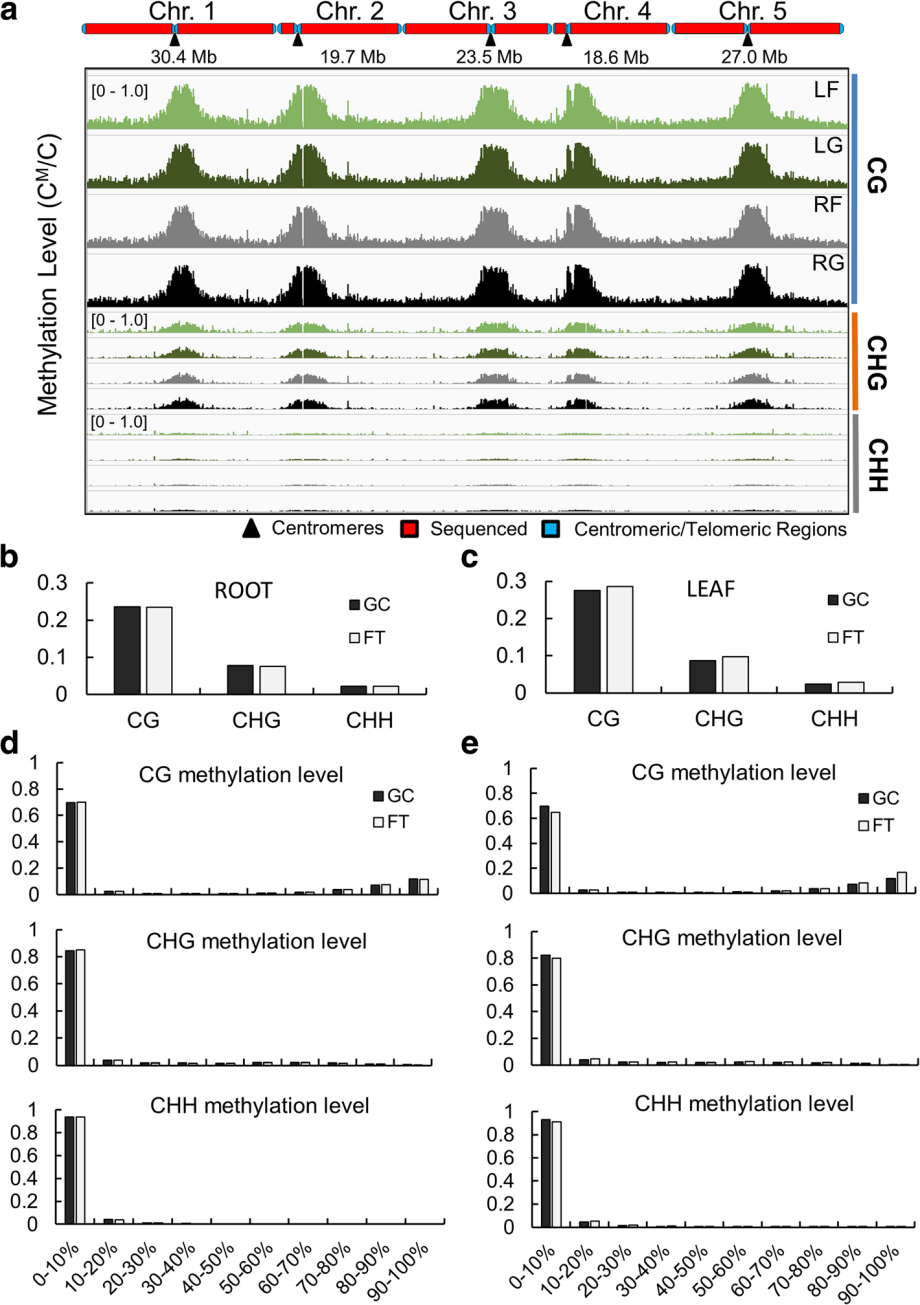
Overview of genome wide DNA methylation levels. **a** Methylation level of CG, CHG and CHH contexts across chromosomes 1–5 in roots and leaves grown in spaceflight (RF and LF) and roots and leaves grown on the ground (RG and LG) were visualized using Integrative Genomics Viewer (IGV) (http://software.broadinstitute.org/software/igv/). Average methylation level of whole genome in root (**b**) and leaf (**c**) samples are shown. The proportion of CG, CHG and CHH contexts in ten individual bins corresponding to different methylation levels are listed for root (**d**) and leaf (**e**) samples

The overall cytosine methylation status within the CG, CHG and CHH contexts in spaceflight samples and ground controls was visualized in the Integrative Genomics Viewer (IGV) (http://software.broadinstitute.org/software/igv/) (Fig. 2a). The general methylation levels and distribution of 5mC across the genome were not altered dramatically in any particular part of the genome nor within any C context (Fig. 2a-c). For each methylation context, total methylation was not obviously changed by spaceflight in roots (Fig. 2b) or leaves (Fig. 2c).

While the average genome-wide methylation levels within each sequence context were not significantly different between spaceflight and ground controls in either organ (Fig. 2b, c), spaceflight leaves did show a slight shift in methylation levels within each methylation context toward more highly methylated sites. Figure 2d and e show the distribution of the proportions of CG, CHC and CHH methylation levels in each organ, comparing the ground control values to spaceflight. For roots (Fig. 2d), there was no obvious difference in the distribution of methylation levels. In leaves there were larger differences between spaceflight and ground control in methylation level distribution (Fig. 2e). Especially for CG context, there was a higher proportion of sites with methylation levels below 10%, and more sites with methylation levels above 90% for spaceflight in leaves. Generally the shift from lower percent methylation to higher percent methylation was observed in all three types of methylation in spaceflight leaves.

### Spaceflight changes in DNA methylation levels were associated with protein-coding genes

**Fig. 3:**
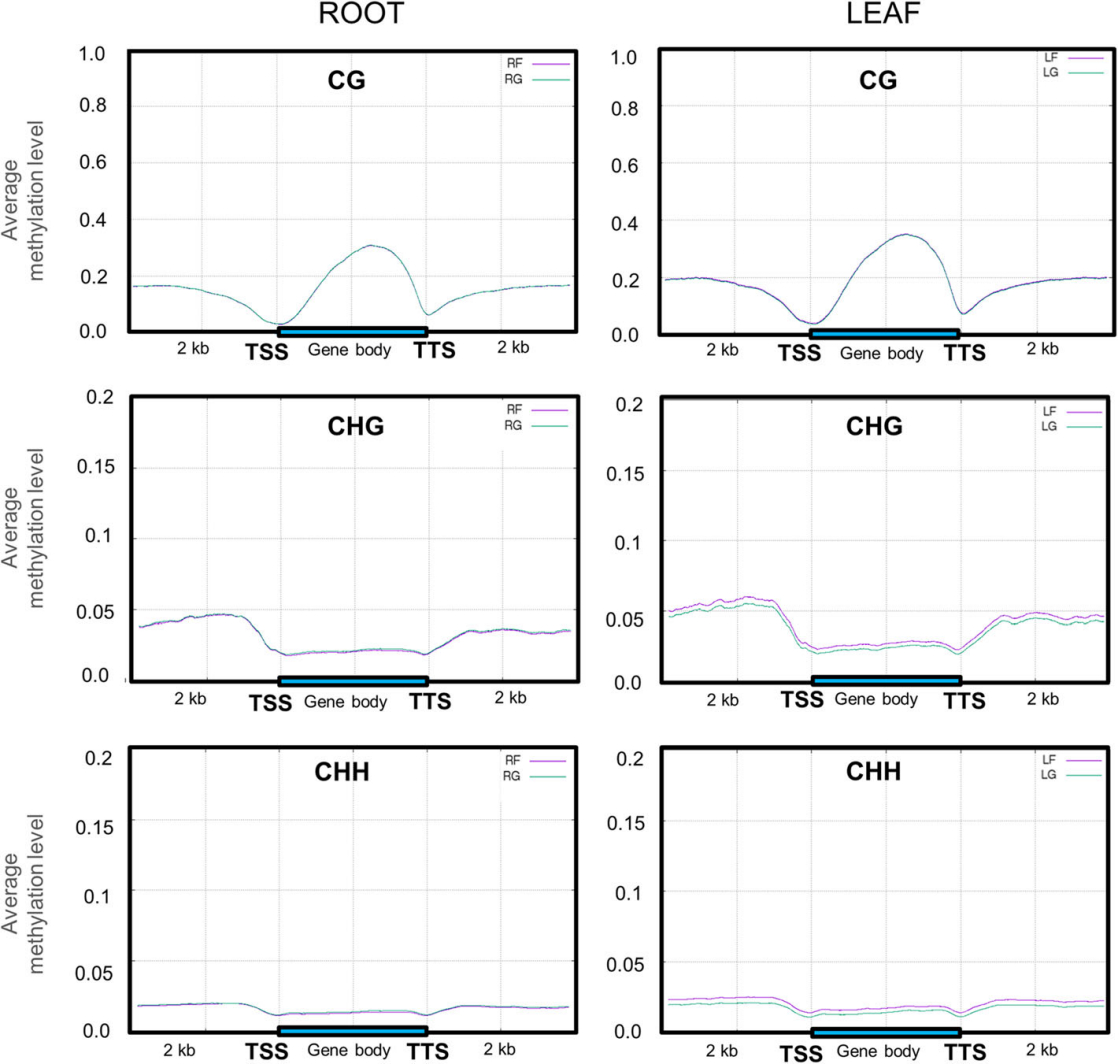
Average methylation levels across protein-coding genes in CG, CHG and CHH contexts. Gene bodies (highlighted by blue bar) as well as flanking regions 2 kb upstream from transcription start site (TSS) and 2 kb downstream from transcription termination site (TTS) are shown in the plots. Roots and leaves grown on the ISS (RF and LF) are indicated by magenta lines, whereas roots and leaves grown on the ground (RG and LG) are indicated by teal lines

Methylation levels were evaluated in the gene bodies (from transcription start site, TSS to transcription termination site, TTS) as well as flanking regions of 2 kb upstream from TSS and 2 kb downstream from TTS for the protein coding genes in Arabidopsis. Figure 3 shows the average methylation levels across protein-coding genes in the CG, CHG and CHH contexts for both roots and leaves. Overall changes in DNA methylation were not associated with any particular region of expressed genes. In roots, methylation levels in CG, CHG and CHH contexts of gene body and downstream regions were virtually identical in ground and spaceflight samples. However, in leaves, CHG and CHH methylation levels across genic regions were increased by spaceflight (Fig. 3). The observed elevation of averaged methylation levels in genes from leaf DNA was largely uniform throughout the gene regions, although the differential in the upstream and downstream regions are slightly more prominent than in the immediate vicinity of transcription start and termination. Thus, although large scale alteration of the average methylation level was not detected on a genome-wide scale, spaceflight influenced CHG and CHH methylation across protein-coding regions in an organ-specific fashion was detected.

### The number of hyper and hypomethylated cytosine sites was influenced by spaceflight especially in leaves

**Fig. 4:**
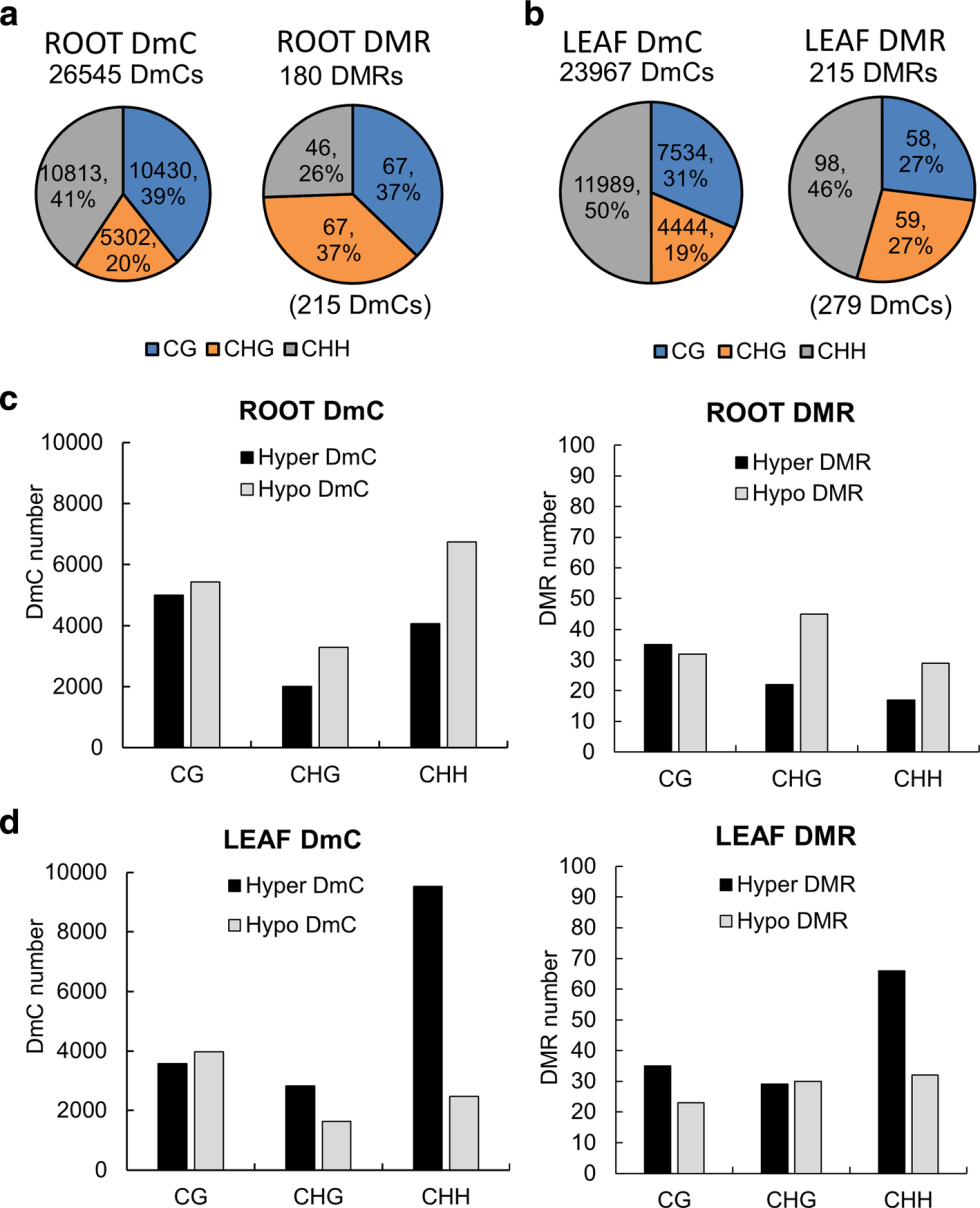
Global analysis of DmCs and DMRs in roots and leaves. Total number of differentially methylated cytosine sites (DmCs), differentially methylated regions (DMRs), number of DmCs within DMRs, and percentage of differential methylation in CG, CHG and CHH contexts of DmCs and DMRs in roots (**a**) and leaves (**b**). Context breakdown of the number of hyper−/hypo- DmCs and DMRs in roots (**c**) and leaves (**d**)

Site-specific differentially methylated cytosines (DmCs) between spaceflight and ground control with statistical significance (FDR < 0.01) were used to evaluate each methylation context genome-wide in both roots and leaves. Additionally, the differentially methylated regions (DMRs) were also used to evaluate the genome-wide methylation context. DMRs were identified by comparing the average methylation levels within a 100 bp window between spaceflight and ground controls and those with statistical significance (FDR < 0.01) were used in the analysis. There were 26,545 DmCs detected in roots in response to spaceflight, 215 of which were also in DMRs (Fig. 4a). In leaves, there were 23,967 spaceflight-associated DmCs, 279 of which were in DMRs (Fig. 4b). These sites in CG, CHG and CHH contexts are listed in Additional file 2: Table S2.

Roots and leaves showed distinct distributions of differentially methylated cytosines. More CG sites and fewer CHH sites were differentially methylated in roots compared with leaves, while proportions of differentially methylated CHG sites were similar in these two organs (Fig. 4a, b). In roots hypomethylated CHH sites predominated, while leaves showed a predominance of hypermethylated CHH sites (Fig. 4c, d). Global hyper/hypomethylation trends between DmCs and DMRs were similar in leaf organs, whereas in roots, hypomethylated CHG DMRs predominated.

### The distribution of gene-related DmCs and DMRs is organ specific

**Fig. 5:**
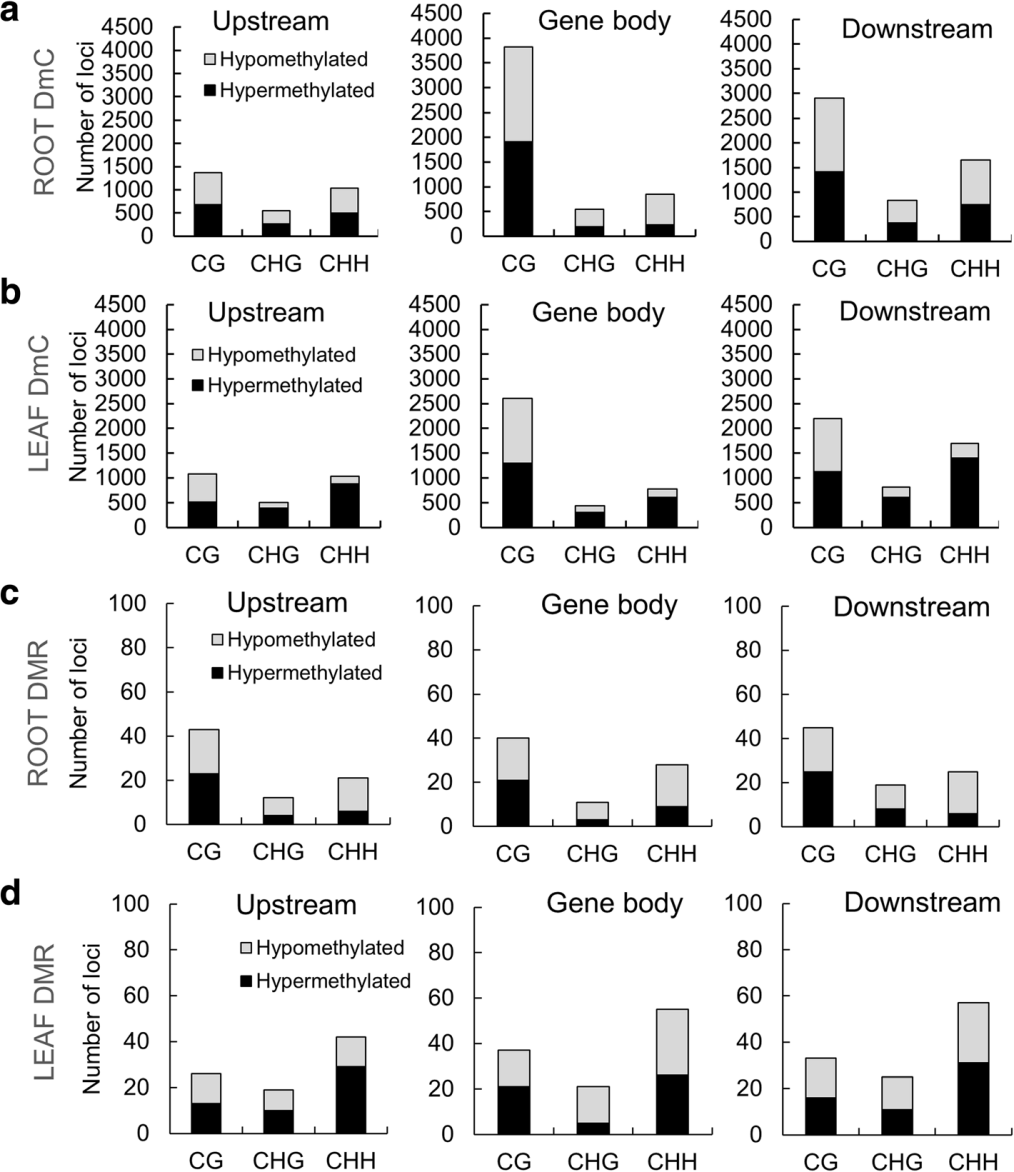
Context breakdown of gene-related DmCs and DMRs. **a-b** Numbers of gene loci affected by hyper- and hypo-DmC in gene bodies, 2 kb upstream from transcription start site, and 2 kb downstream from transcription termination site in response to spaceflight. **c**-**d** Numbers of gene loci affected by hyper and hypo-DMRs are mapped to the gene-associated regions described above

The distributions of DmCs and DMRs within gene regions varies between roots and leaves, as do the proportions of hypo- and hypermethylated cytosines among CG, CHG and CHH contexts. Figure 5 shows the context of gene-related DmCs and DMRs within regions 2 kb upstream from transcription start site, and 2 kb downstream from transcription termination site. The numbers of gene loci that are affected by hyper- and hypo-DmC and DMRs in gene bodies for roots and leaves in response to spaceflight are shown in Fig. 5a, b, and the numbers of gene loci affected by hyper and hypo-DMRs in Fig. 5c, d for each organ. Gene-related DmCs in roots and leaves were predominantly positioned in the gene body within the CG context. Gene-related DMRs in roots were evenly distributed across all three gene structures predominantly in the CG context. Whereas gene-related DMRs in leaves were distributed across all three gene structures predominantly in the CHH context (see also Additional file 3: Table S3).

### Correlation of differential DNA methylation and differential gene expression

**Fig. 6:**
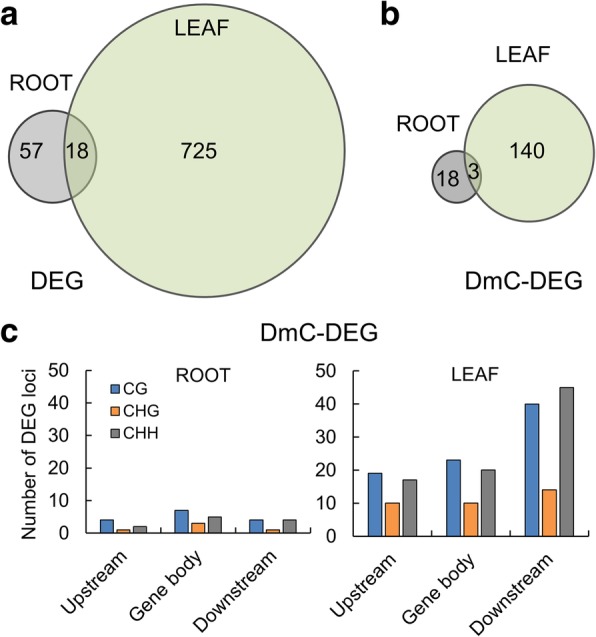
Relationship between DEGs and gene-related DmCs. **a** Venn diagram of differentially expressed genes (DEGs) overlapped in roots and leaves. Eight hundred DEGs in roots or leaves between spaceflight and ground control had statistical significance (FDR < 0.05) with a fold-change of at least 2. **b** Venn diagram of the number DEGs mapped with DmCs in roots and leaves. **c** Numbers of DEGs in root and leaf that mapped with DmC in each methylation context of the various gene structures (2 kb upstream of TSS, gene body, and 2 kb downstream of TTS)

RNA-seq was used to establish organ-specific, differential patterns of gene expression between plants grown in the spaceflight environment and their comparable ground controls. Hierarchical clustering and principal component analysis (PCA) show that the two organs evaluated, roots and leaves, have widely different patterns of gene expression, which highlighted the value of separating the two organs in the evaluation of the spaceflight response (Additional file 7: Figure S1 , Additional file 8: Figure S2 and Additional file 9: Figure S3). There were 800 genes differentially expressed with statistical significance (FDR < 0.05) by at least 2-fold in roots or leaves, in the comparison of spaceflight vs ground control (Additional file 4: Table S4). Among them, 75 differentially expressed genes were detected in roots while 743 were differentially expressed in leaves, with 18 genes shared between the two tissues (Fig. 6a). Similar with the results of the previous *Arabidopsis* transcriptome survey from the ISS [14], the genes associated with temperature stresses, oxidative stimuli, and biotic stress were widely influenced (Additional file 4: Table S4). Of the genes differentially expressed in response to spaceflight, 143 DEGs from leaves, and 21 DEGs from roots were among those genes which were also differentially methylated in response to spaceflight (Fig. 6b). Figure 6c shows the numbers of DEGs in root and leaf that mapped with DmCs among CG, CHG and CHH contexts in the various gene structures (2 kb upstream of the transcription start site, the gene body, and 2 kb downstream of the transcription termination site).

**Fig. 7:**
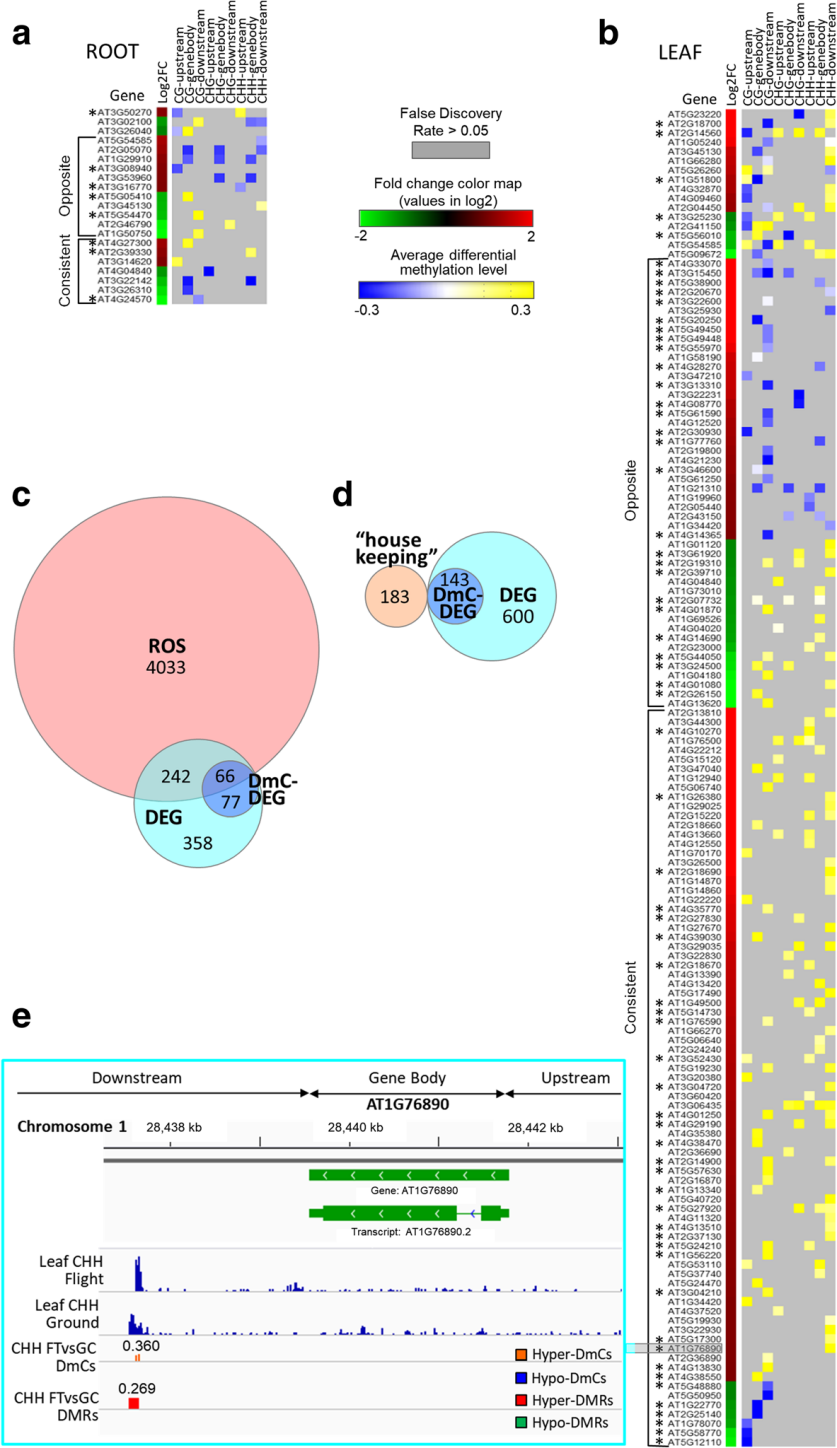
Correlation of differential gene expression and DNA methylation in roots and leaves show ROS association. Heatmap representation of differential gene expression (Log2 fold-change) and differential DNA methylation (average level in area indicated; Gene body: TSS to TTS, upstream: 2 kb from TSS, downstream: 2 kb from TTS) of 21 genes in roots (**a**) and 143 genes in leaves (**b**). Opposite trends are indicative of upregulated genes with hypomethylated DmCs or downregulated genes with hypermethylated DmCs whereas a consistent trend shows the converse relationship. ROS associated genes are indicated with an asterisk. **c** Venn diagram shows a visual representation of spaceflight DEGs and DmC-associated DEGs that overlap with ROS genes. **d** Venn diagram shows a visual representation of spaceflight DEGs and DmC-associated DEGs that overlap with housekeeping genes. **e** Visualization of the methylation data using the Integrative Genomics Viewer (IGV) (http://software.broadinstitute.org/software/igv/). The full–genome methylome profiling in the CHH context of spaceflight (Flight) and ground control (Ground) leaf sample along with DmC and DMR data in the corresponding region

Figure 7 provides a heat map of the complete list of DmC-DEG genes, and summarizes relevant features of this group. For each gene from roots (Fig. 7a) and leaves (Fig. 7b), the gene ID number is aligned with the differential expression heat map and an indication of the average differential methylation level for CG, CHG and CHH contexts in respective regions (Fig. 7a, b; Additional file 5: Table S5). The consistent or opposite trend between differential expression and DNA methylation is also indicated in the figure. The opposite trend is indicative of upregulated genes with hypomethylated DmCs or downregulated genes with hypermethylated DmCs of all three types of methylation in the genic regions whereas the consistent trend shows the converse relationship. In roots, 11 genes showed opposite trend and 7 genes showed consistent trend (Fig. 7a). Gene ontology analysis detected stress responses to abiotic stimulus, temperature, radiation, and light stimulus. In leaves, 48 genes showed opposite trend and 79 genes showed consistent trend (Fig. 7b). Gene ontology analysis also detected response to stressors of temperature, radiation and light, plus chemical stress, oxidative stress, and a response to oxygen-containing compounds (Additional file 5: Table S5). The correlation of gene expression and DNA methylation in a certain type was also reflected by scatterplots using genes with differential expression as well as differential DNA methylation of CG, CHG and CHH contexts in respective gene regions. In general, CG methylation showed a strong negative correlation with gene expression in roots, while CHH hypermethylation was positively correlated to gene upregulation in leaves (Additional file 10: Figure S4).

Genes associated with reactive oxygen species (ROS) signaling were well represented in the DmC-DEG sets (starred gene ID numbers, Fig. 7a, b), particularly in leaves, where ROS genes comprised 46% of the differentially methylated, differentially expressed genes (DmC-DEG) (Additional file 6: Table S6). In both roots and leaves, ROS genes appeared about equally distributed between both negative and positive correlations of differential expression and DNA methylation. The Venn diagram [76] shown in Fig. 7c provides a visual representation of these data: 4341 ROS genes [77] were compared to the 743 leaf DEGs and the 143 leaf DmC-DEGs. A slightly smaller percentage (41%) of the total leaf spaceflight DEGs were also classified as ROS (Fig. 7c). The spaceflight-associated DmC-DEGs also participate in terrestrially-characterized ROS signaling induced by various conventional stressors including reactive oxygen species, high light, UV, pathogen associated molecules, and oxidation related mutation, etc. Mapping of the ROS related stress category to the ROS-associated genes differentially expressed in spaceflight did not suggest an obvious correlation with any specific category, but in general, there appeared to be a somewhat negative association with ROS stress categories I, II and III, and a somewhat positive association with ROS stress categories IV through VIII (Additional file 6: Table S6). For contrast, the leaf spaceflight DEGs and DmC-DEGs were compared to a set of genes identified as reference, or “housekeeping” genes [78]; none of the 183 reference genes were represented in either the DEG or DmC-DEG sets (Fig. 7d). One of the ROS genes is presented to show the distribution of differential methylation using the IGV browser graphics (Fig. 7e). The spaceflight-associated hypermethylation of ROS-associated transcription factor GT2 (AT1G76890) predominated in the downstream region of the gene in the CHH context.

## Discussion

When subjected to spaceflight aboard the ISS, plants engage changes in DNA methylation in addition to the now well-characterized changes that occur in gene expression. This conclusion is consistent with the notion that some aspects of gene regulation networks are controlled at the epigenetic level for terrestrial plant stresses and ecological conditions, and suggests that spaceflight physiological adaptation in plants likely involves DNA methylation.

DNA methylation has been shown to be associated with many critical aspects of molecular regulation, such as gene and transposon silencing, genomic imprinting as well as X chromosome inactivation [79–83]. Given that epigenetic modifications have been demonstrated to play a key role in gene regulation in multiple systems [84, 85], DNA methylation likely presents one mechanism whereby an organism takes an environmental cue and tangibly alters its genetic blueprints to respond to that cue, record that genomic alteration, and potentially pass that information on to its progeny [71, 86, 87]. Despite many demonstrations that DNA methylation is part of terrestrial environmental responses, few DNA methylation studies have been performed in the context of the spaceflight environment. The literature on spaceflight-associated epigenomic studies of plants has thus far included analyses of plants grown on Earth from seeds subjected to the spaceflight environment and seedlings grown on Earth and then subjected to 60 h of spaceflight in a satellite [88]. In the seed exposure experiment, analyses with methylation-sensitive restriction enzymes revealed that the methylation state of 11 genes and 6 transposable elements (TEs) in terrestrially-grown rice plants from seeds flown on the “Long-March-2” spaceship for 18 days was altered compared to the control plants grown from seed that was not exposed to spaceflight [89–92]. In recent experiments with seedlings grown on the ground for 6 days then exposed to spaceflight on board the SJ-10 recoverable satellite for 60 h [88], whole seedlings also demonstrated differential DNA methylation between spaceflight and ground controls.

The work presented here represents the first gene-expression correlated, organ specific, whole genome survey of DNA methylation in plants that were sent to space as seeds, then germinated and grew in space under conditions anticipated for plant production in support of human exploration. These ISS grown plants were harvested and preserved by astronauts on orbit, and the frozen samples were returned to Earth for a direct comparison of ISS-grown and Earth-grown leaves and roots. The methylome and correlated transcriptome results from these 11 day old plants strongly reinforce the distinct organ-specific nature of the spaceflight response in that the spaceflight responses in gene expression and DNA methylation were different between roots and leaves. An interesting feature of these data is that while the physiological adaptation to spaceflight did not include large scale, genome wide changes in DNA methylation, the methylation levels within each context shifted toward more highly methylated sites in spaceflight leaves, which changed the distribution of the proportions of CG, CHC and CHH methylation levels. So in leaves, but not roots, the average methylation levels across protein-coding genes changed within each CG, CHG and CHH context. Specific differential methylations were observed in locations relevant to gene expression. The general notion of spaceflight-induced changes in DNA methylation reported here support the overall conclusion obtained with whole seedlings grown on Earth then exposed to a 60 h satellite spaceflight [88]; however, Xu et al. (2018) observed wide scale hypomethylation in spaceflight, a phenomenon that was not observed in the present study of plants that germinated and lived entirely on the ISS. In addition, a number of genetic mutations were observed in rice seedlings experiencing 18 day of spaceflight in satellites [90, 92], whereas we did not detect any evidence of mutation in the genome of this set of *Arabidopsis*. The source of such distinctions between experiments can be attributed to many factors, including the degree of internal shielding (ISS vs. a satellite), the growth hardware and internal environment, as well as the age and growth strategy of the plants.

It has been reported that hypermethylation in promoters is generally related to repressed transcription levels, especially in that CG methylation near TSS and TTS negatively affects gene expression [93, 94]. However, in the spaceflight data presented here, both positive and negative correlations were observed between CG methylation and gene expression in leaves (Additional file 10: Figure S4). Meanwhile, CHH hypermethylation showed a strong correlation to gene upregulation in leaves but not roots, demonstrating the organ-specific molecular regulatory network. In addition, both positive and negative correlations were observed with differential transcription and differential methylation across genic regions in all three contexts. These indicate that any correlation between DNA methylation and gene expression of plants in spaceflight is neither simple nor limited to a specific cytosine context or location with respect to gene structure, though some general trends can be identified in an organ-specific manner. The employment of DNA methylation associated mutant materials in future spaceflight experiments may further clarify the functional relationship between gene expression regulation and different types of DNA methylation.

In leaves, about one fifth of the genes that were significantly differentially expressed by at least two-fold were among those genes that also showed differential methylation, and in roots the fraction was about one third; there were 21 of 75 differentially expressed genes in roots and 143 of 743 differentially expressed genes in leaves that were also differentially methylated. This result suggests that DNA methylation may play a significant role in a portion of the genes regulated by spaceflight, although DNA methylation is clearly not the only factor in the regulation of gene expression in response to spaceflight.

DNA methylation occurs with great density at repetitive sequences such as the pericentromeric regions, and in light density in euchromatin [95]. The non-coding regions flanking active genes can participate the regulation of transcription initiation and abundance [96]. In Arabidopsis, CG methylation is mainly controlled by methyltransferase MET1 and chromatin remodeler DDM1, while CHG and CHH methylation mainly relies on the methyltransferases CMT3 and DRM2 [97]. DRM2 dependent non-CG methylation is guided by small RNAs [97]. In our data sets no known RNA-directed DNA methylation (RdDM) pathway genes were differentially expressed. Whereas the increased CHG and CHH methylation levels in coding sequences as well as flanking regions in leaves experiencing spaceflight suggest a possibility that the ISS environment elicits organ-specific effects in CMT3 and/or DRM2-dependent functions in protein-coding genic regions. Moreover, DNA methylation is correlated with genome stability especially in transposable elements [98]. This initial comprehensive investigation uncovered the organ-specific correlation between DNA methylation and gene expression in genes involved in many classes of abiotic stress genes previously associated with a spaceflight response, including representatives of oxidative stress, heat shock, cell wall remodeling and defense signaling [14]. The auxin related genes involved in gravitropism, such as *SAUR* and *GH3* families, were also altered. However, the most prominent category of genes represented in those that are both differentially expressed and differentially methylated in response to spaceflight are those associated with ROS signaling. ROS signaling genes cross into many other metabolic categories, and a recent meta-analysis characterized the extent of how oxidative stress reactions shaped transcriptomic responses, and referred to this complex of ROS-associated genes as the ROS Wheel [77]. Cross referencing the ROS Wheel genes with the spaceflight DmC-DEGs from leaves revealed 46% overlap (Fig. 7, Additional file 6: Table S6), which is slightly higher than the proportion of ROS genes in spaceflight DEGs overall (41%). Cross referencing the ROS Wheel genes with other stress-associated transcriptomes, such as osmotic stress (34%) [99], and pathogen stress (30%) (SUBA3 - http://suba3.plantenergy.uwa.edu.au/ [100]), reveals that a smaller proportion of these stresses are associated with ROS signaling. In addition, a number of ROS related genes behave in a diverse manner in spaceflight compared with the responses to conventional stresses such as high light exposure (Additional file 6: Table S6). These data suggest that ROS genes may play a unique role in the physiological adaptation of plants to the spaceflight environment.

## Conclusions

The *Arabidopsis thaliana* seedlings grown in the Veggie hardware on the ISS showed organ-specific differences in gene expression and DNA methylation as compared with the ground controls. Although the overall methylation levels were not significantly changed by spaceflight, thousands of spaceflight differentially methylated cytosine sites were detected in both roots and leaves. Spaceflight leaves showed higher methylation levels in CHG and CHH contexts within protein-coding genes compared with ground control. Moreover, dozens of genes exhibited negative or positive correlation between differential expression and differential methylation in roots and leaves. Over 46% of the differentially expressed and differentially methylated genes were associated with ROS signaling. The work presented here represents the first gene-expression correlated, organ specific, whole genome survey of DNA methylation in plants that were sent to space as seeds, then germinated and grew in space. These data provide fundamental insight into the strategies plants use in the physiological adaptation to the novel environment presented by spaceflight.

## Methods

The data presented here are part of the spaceflight experiment Advanced Plant Experiment 03–2 (APEX03–2) which was launched on SpaceX mission CRS-5 on 10 January 2015 in a Dragon Capsule carried by a Falcon 9 rocket departing from Complex 40 at Kennedy Space Center. This experiment is also identified by NASA operations nomenclature (OpNom) as “TAGES-ISA” (Transgenic *Arabidopsis* Gene Expression System-Intracellular Signaling Architecture: http://www.nasa.gov/mission_pages/station/research/experiments/1059.html). The component of the TAGES-ISA experiment described here comprises a single genotype of Arabidopsis grown for 11 days in on the ISS.

### Plant material and plate preparations

Dry-sterilized Arabidopsis seeds of the Wassilewskija (WS) ecotype were planted onto the surface of 100 mm^2^ square petri dishes containing a solid nutrient medium of 0.5% Phytagel/0.5x MS (e.g. [14, 41]). Each plate was planted with 12 to 15 seeds. The plates were immediately wrapped in black cloth (Duvetyne, SeattleFabrics.com) to help preserve seed dormancy [101]. The wrapped plates were stored at 4 °C until launch. Three plates of WS seeds were launched for this part of the experiment, and there were three comparable ground control plates. The seeds remained dormant until removal from cold stowage and exposure to light after installation of the plates into the Vegetable Production System (VPS, aka “Veggie”) hardware on the Columbus Module of the ISS. This WS Arabidopsis seed line has been propagated in our laboratory for about 25 years; the WS line is curated as ABRC stock CS915 (www.arabidopsis.org). Examples of previous experiments using this seed line include these relevant studies [7, 8, 14, 18, 76, 78]. Samples of the seed used in this study are available for distribution upon request.

### Plant growth conditions on ISS and ground controls

To initiate germination, plates were unwrapped and secured perpendicularly to the LED light source of the Veggie hardware, which generated constant light conditions of 100–135 μmoles/m^2^/s PAR (Fig. 1a). The plants were grown for 11 days and individual plates were subsequently removed from Veggie and harvested by astronaut CDR Butch Wilmore into Kennedy Space Center (KSC) Fixation Tubes (KFTs) and fixed in RNAlater™ (Ambion, Grand Island, NY, USA). KFTs provide three levels of liquid containment and are quintessential ISS hardware for studying many aspects molecular biology (e.g. [9, 14, 41, 102–104]). One plate of plants was harvested to an individual KFT. The three KFTs were then stowed at − 80 °C in the MELFI freezer aboard the ISS, and remained frozen during the return to Earth for de-integration at KSC. The frozen samples were transferred from KFTs to 50 mL falcon tubes, and remained frozen until delivery to the Principal Investigators for analysis.

The corresponding ground controls were grown in the ISS Environmental Simulation (ISSES) chamber at KSC on a 24 h delay. The delay in initiation of the ground control was to facilitate programing the ISSES chamber with the ISS environmental profile of temperature, CO_2_ levels and lighting. The ground controls were unwrapped, placed into the Veggie ground unit within the ISSES chamber, and grown for 11 days in an identical fashion to the spaceflight samples. The plants were then harvested to KFTs (one plate to tone KFT) and stored in a standard − 80 °C freezer by an astronaut surrogate. The ground KFTs were kept frozen for the same amount of time as the spaceflight samples, and then the frozen samples in each KFT were transferred to individual 50 mL falcon tubes, and remained frozen until delivery to the Principal Investigators for analysis.

### Plant dissection, RNA and DNA extraction

The 50 mL Falcon tubes, each containing the plants from one plate preserved in RNAlater, were thawed and gradually brought to room temperature; plants were submerged in RNAlater at all times. Each plate represented a biological replicate comprising 12–15 individual 11 days old plants (Fig. 1b). From each replicate, three individual plants were pooled for RNA extraction and the remainder (9–12 plants) were pooled for genomic DNA extraction. Leaves, hypocotyls and roots from each set were dissected on an Olympus dissecting microscope into to leaf and root samples (Fig. 1c). The intervening hypocotyls were set aside. RNA extraction was performed using RNeasy Plant Mini Kit (Qiagen, Hilden, Germany) and the quality and quantity of the RNA samples were assessed using Agilent 2100 Bioanalyzer (Agilent Technologies, Inc.). DNA was extracted using a modified phenol/chloroform protocol as previously described [105], as residual components from the RNAlater preservation interfered with spin-column based gDNA kits. The quality and quantity were determined using a DNA1200 BioAnalyzer chip (Agilent Technologies, Santa Clara, CA, USA). Three biological replicates of root and leaf samples from spaceflight and ground controls were used for both RNA-seq and WGBS.

### RNA sequencing and bisulfite DNA sequencing

RNA extraction was performed using RNeasy Plant Mini Kit (Qiagen, Hilden, Germany) according to the manufacturer’s guidelines RNA concentration was determined on Qubit® 2.0 Fluorometer (ThermoFisher/Invitrogen, Grand Island, NY), RNA quality was assessed using the Agilent 2100 Bioanalyzer (Agilent Technologies, Inc.). RNAseq libraries were constructed using NEBNext Ultra Directional RNA Library Prep Kit for Illumina (NEB, USA) for poly(A) mRNA isolation following manufacturer’s recommendations. Whole Genome Bisulfite Sequencing was performed similarly to previous experiments [106]. In brief, 700–1000 ng of genomic DNA was processed for sequencing library construction. The DNA was transferred into 6 × 16 mm glass microtubes with AFA fiber and pre-slit snap caps and sheared into average fragments of ~ 200 bp using the Covaris S2 ultrasonic disruptor, following the manufacturer settings. Fragments of less than 100 bp in size were removed using AMPure magnetic beads (Beckman Coulter, Brea, CA, USA) at a 1:1 bead to sample ratio and 300–750 ng of clean, fragmented DNA was obtained. Between 100 and 250 ng of this material was used for Illumina sequencing library construction using the NEBnext Ultra DNA Illumina construction kit (New England Biolabs, Ipswich, MA, USA) in conjunction with Illumina-specific methylated and barcoded adaptors (New England Biolabs). After end-repair, 3′-adenylation, and adaptor-ligation steps, 75–150 ng of clean, adaptor-ligated DNA fragments (Illumina library) were size-selected to 250–500 bp (average ~ 320 bp) using AMpure beads and recovered in Tris-HCl, pH 8.0 with quantification using a QUBIT fluorimeter (Life Technologies, Carlsbad, CA, USA). Likewise, libraries were bisulfite converted using the EZ DNA Methylation™ Kit (Zymo Research, Irvine, CA, USA) according to the manufacturer’s instructions. The resulting bisulfite converted libraries were enriched by an amplification with 15 cycles using a uracil-insensitive polymerase (NEB) prior to purification with AMPure beads. The final processed libraries were quantified by the QUBIT fluorometer and by qPCR with the Kapa SYBR Fast qPCR reagents (Kapa Biosystems) with monitoring on an AB17900HT real-time PCR system (Life Technologies). Twelve barcoded bisulfite-seq libraries and 12 barcoded RNA-seq libraries (with triplicates for each condition) were pooled with the ratio of RNA: DNA = 60%: 40% and sequenced on three flow cell lanes on the HiSeq3000 instrument using a 2 × 101 cycle multiplex paired-end reads per lane.

### Reads alignment and processing

The quality of the RNA-Seq sequence data was first evaluated using FastQC prior to further downstream analysis. Low quality sequences were removed and poor quality part of the reads were trimmed using Trimmomatic [107]. The Star Aligner [108] was used to map high quality paired-end reads to TAIR10 genome. Gene expression was obtained using RSEM [109]. The expected read counts and Fragments Per Kilobase of transcript per Million mapped reads (FPKM), were extracted for further analysis. A generalized linear regression model was built to perform the differential gene analysis using edgeR [110]. The criteria that Counts per Million mapped reads (CPM) above 5 in at least 3 samples out of all RNA libraries was used to gate genes with extremely low expression. Prior to the differential analysis, hierarchical clustering and Principal Component Analysis (PCA) were conducted to identify the potential outlier of the samples. The thresholds for calling significantly differential expressed genes were set at FDR 0.05, the fold change of greater than 2, and the average FPKM for at least one of each comparison group is higher than 0.

The input bisulfite-seq reads were trimmed using trimmomatic and quality control was performed before and after trimming using FastQC. Reads were aligned to the *Arabidopsis thaliana* (WS) reference genome using bsmap [111]. Methylation calling was performed with cscall v1.0 [112] using the ws_0.v7.allPlusChlMito-CG/CHG/CHH index (Cscall Available at: http://compbio.ufl.edu/software/cscall/; A. Riva, Gainesville, FL: UF Computational Biology). A cytosine site was included in the analysis if it reached at least 10 x read coverage in at least 2 replicates for each condition. Differential methylated cytosine (DmCs) were identified and determined using the mcomp program [113, 114]. Cytosines in CG, CHG or CHH contexts for which the FDR (False Discovery Rate) corrected *p* value of the difference between test and control methylation rates was below 0.01 were considered significant. The differentially methylated regions (DMR) were identified using the method previously described [84]. In brief, a DMR was defined as a window of 100 bp containing at least 4 DmCs, with a methylation difference of at least 0.2 and a significance level of 0.01. DMRs separated by not more than one window were joined together in a larger DMR. DmCs and DMRs were also categorized based on the characteristics of their genomic locations including gene body (from transcriptional start site to transcriptional terminate site), promoter (2 kb upstream of transcriptional start site) and downstream (2 kb downstream of transcriptional terminate site). The reads mapped to chloroplast reference genome were used to calculate the ratio of converted cytosines in CG, CHG and CHH contexts, which showed the bisulfite conversion efficiency.

### Gene ontology analysis

Gene ontology annotation was performed using agriGO v2.0 [115], a public online tool and database (http://systemsbiology.cau.edu.cn/agriGOv2/index.php). The significance level for GO terms is FDR < 0.05.

## Additional files

Additional file 1: **Table S1.** The bisulfite conversion efficiency based on conversion ratio of cytosines in chloroplast reference genome. The ratio of converted cytosines in CG, CHG and CHH contexts were calculated using reads mapped to chloroplast reference genome. (XLSX 9 kb)

Additional file 2: **Table S2.** The list of differentially methylated cytosines (DmCs) with statistical significance FDR < 0.01) in the CG, CHG or CHH contexts in roots and leaves in the comparison between spaceflight and ground control. (XLSX 2276 kb)

Additional file 3: **Table S3.** The list of genes mapped with differentially methylated cytosines (DmCs) with statistical significance (FDR < 0.01) in the comparison between spaceflight and ground control. DmCs mapped to the location of upstream, gene body and downstream of the gene structure in CG, CHG or CHH contexts in roots and leaves were shown. (XLSX 1491 kb)

Additional file 4: **Table S4.** The list of 800 genes that were differentially expressed with statistical significance (FDR < 0.05) by at least 2-fold in roots or leaves in the comparison between spaceflight and ground control. Genes were categorized by differential expression levels. The Log2 fold-change values with FDR < 0.05 were highlighted with yellow. Red indicates up-regulation and blue indicates down-regulation. (XLSX 97 kb)

Additional file 5: **Table S5.** The list of differentially expressed genes with statistical significance (FDR < 0.05) by at least 2-fold in roots or leaves mapped with differentially methylated cytosines (DmCs) with statistical significance (FDR < 0.01) in the comparison between spaceflight and ground control. Log2 fold-change of expression levels and average differential methylation levels in CG, CHG or CHH contexts in the location of upstream, gene body and downstream of the differentially expressed genes in roots and leaves, and the correlation between gene expression and DNA methylation were shown. GO terms of biological process (FDR < 0.05) were listed for genes in roots and leaves. (XLSX 25 kb)

Additional file 6: **Table S6.** The list of differentially expressed and differentially methylated genes associated with the ROS signaling genes identified by Willems et al. 2016 [77]. The ROS genes with at least 2-fold expression change in response to at least one stress category were selected. (XLSX 26 kb)

Additional file 7: **Figure S1.** Multidimensional scaling (MDS) plots of DNA methylation levels for all 12 DNA samples from roots and leaves of spaceflight and ground control with 3 biological replicates. (TIF 1812 kb)

Additional file 8: **Figure S2.** Principal Component Analysis (PCA) of the whole transcriptome for all 12 RNA samples from roots and leaves of spaceflight and ground control with 3 biological replicates. (TIF 1892 kb)

Additional file 9: **Figure S3.** Frequencies of CG, CHG, and CHH sites in the *A. thaliana* genome. (TIF 198 kb)

Additional file 10: **Figure S4.** The correlation of differential gene expression and DNA methylation changes between spaceflight and ground control in CG, CHG, and CHH contexts. The scatterplots were generated using Log2 fold-change of expression levels and average differential methylation levels in CG, CHG or CHH contexts in the location of upstream, gene body and downstream of the differentially expressed genes in roots and leaves. The data can be located in Additional file 5: Table S5. (TIF 702 kb)

## Abbreviations

ABRS: Advanced Biological Research System
APEX: Advanced Plant Experiment
Bisulfite-seq: Bisulfite sequencing
DEG: Differentially Expressed Genes
DmC: Differentially methylated Cytosine
GO: Gene ontology
IGV: Genomics Viewer
ISS: International space station
ISSES: ISS Environmental Simulation
KFTs: Kennedy Space Center Fixation Tubes
RNA-seq: RNA sequencing
TAGES-ISA: Transgenic Arabidopsis Gene Expression System-Intracellular Signaling Architecture
TSS: transcription start site
TTS: transcription termination site
WGBS: Whole Genome Bisulfite Sequencing
WS: Wassilewskija
