# Supplementary figures and images for: Epigenomics in an extraterrestrial environment: organ-specific alteration of DNA methylation and gene expression elicited by spaceflight in Arabidopsis thaliana
Source: BMC Genomics. 2019 Mar 12;20:205. doi: 10.1186/s12864-019-5554-z (PMC6416986; doi:10.1186/s12864-019-5554-z)

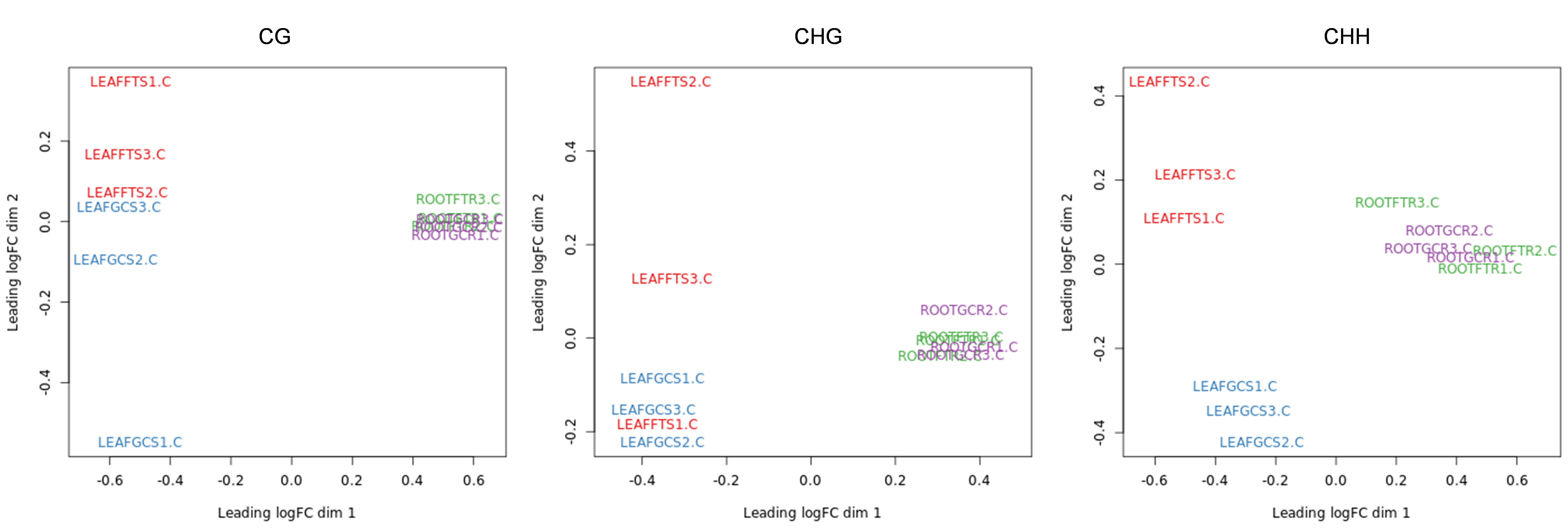

Supplement: Supplementary file 7 — Figure S1. Multidimensional scaling (MDS) plots of DNA methylation levels for all 12 DNA samples from roots and leaves of spaceflight and ground control with 3 biological replicates. (TIF 1812 kb) [file 12864_2019_5554_MOESM7_ESM.tif]

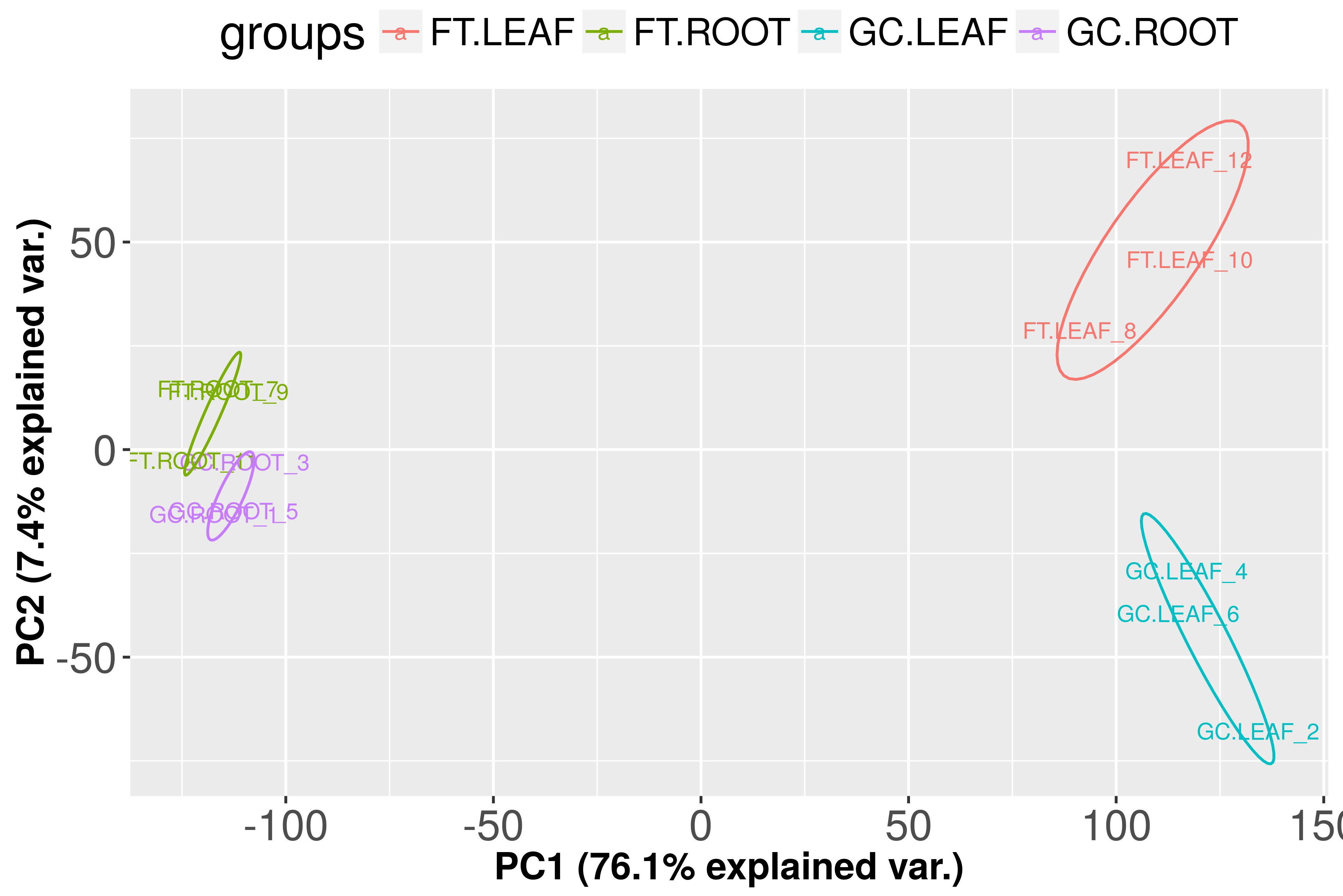

Supplement: Supplementary file 8 — Figure S2. Principal Component Analysis (PCA) of the whole transcriptome for all 12 RNA samples from roots and leaves of spaceflight and ground control with 3 biological replicates. (TIF 1892 kb) [file 12864_2019_5554_MOESM8_ESM.tif]

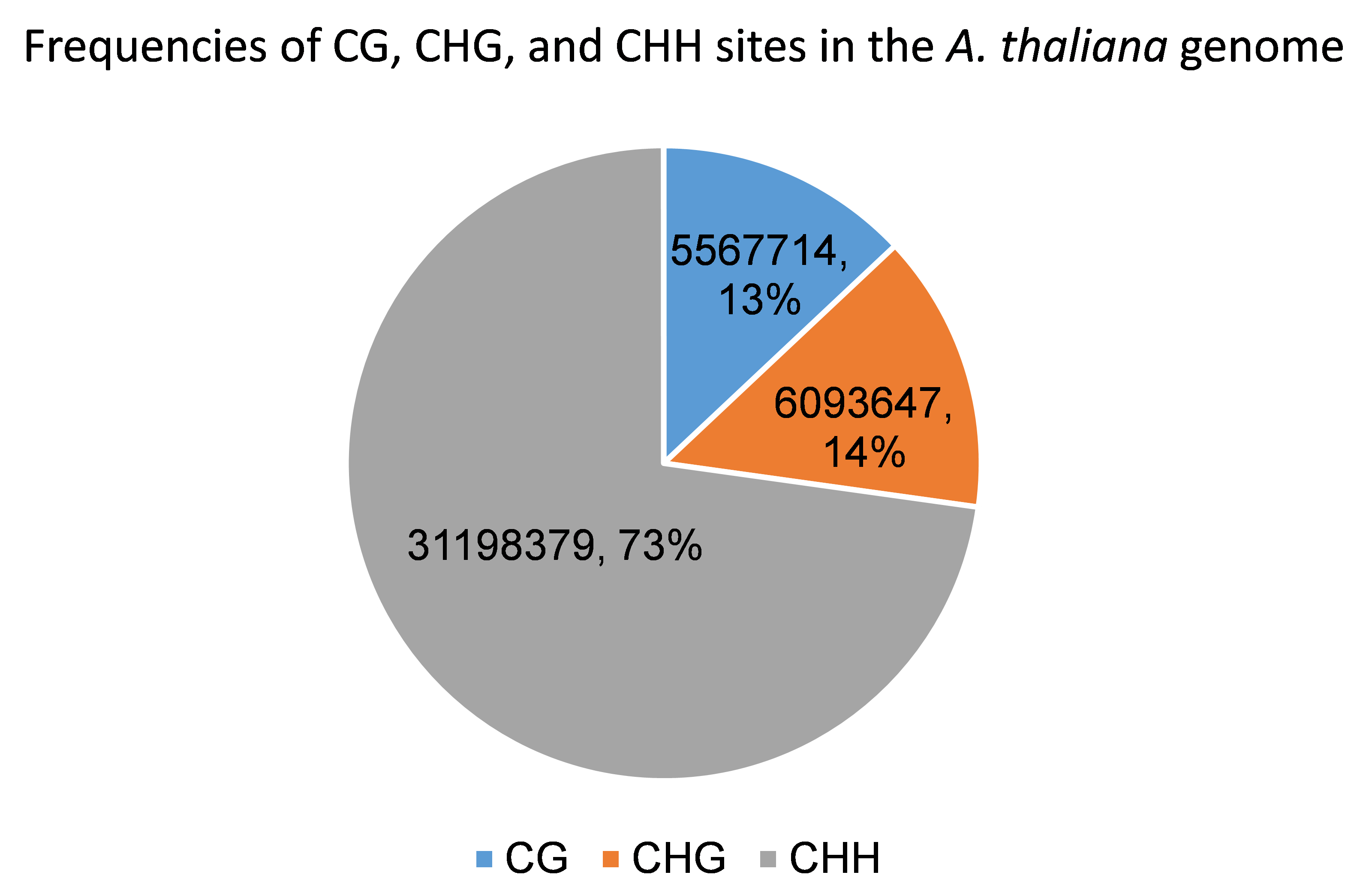

Supplement: Supplementary file 9 — Figure S3. Frequencies of CG, CHG, and CHH sites in the A. thaliana genome. (TIF 198 kb) [file 12864_2019_5554_MOESM9_ESM.tif]

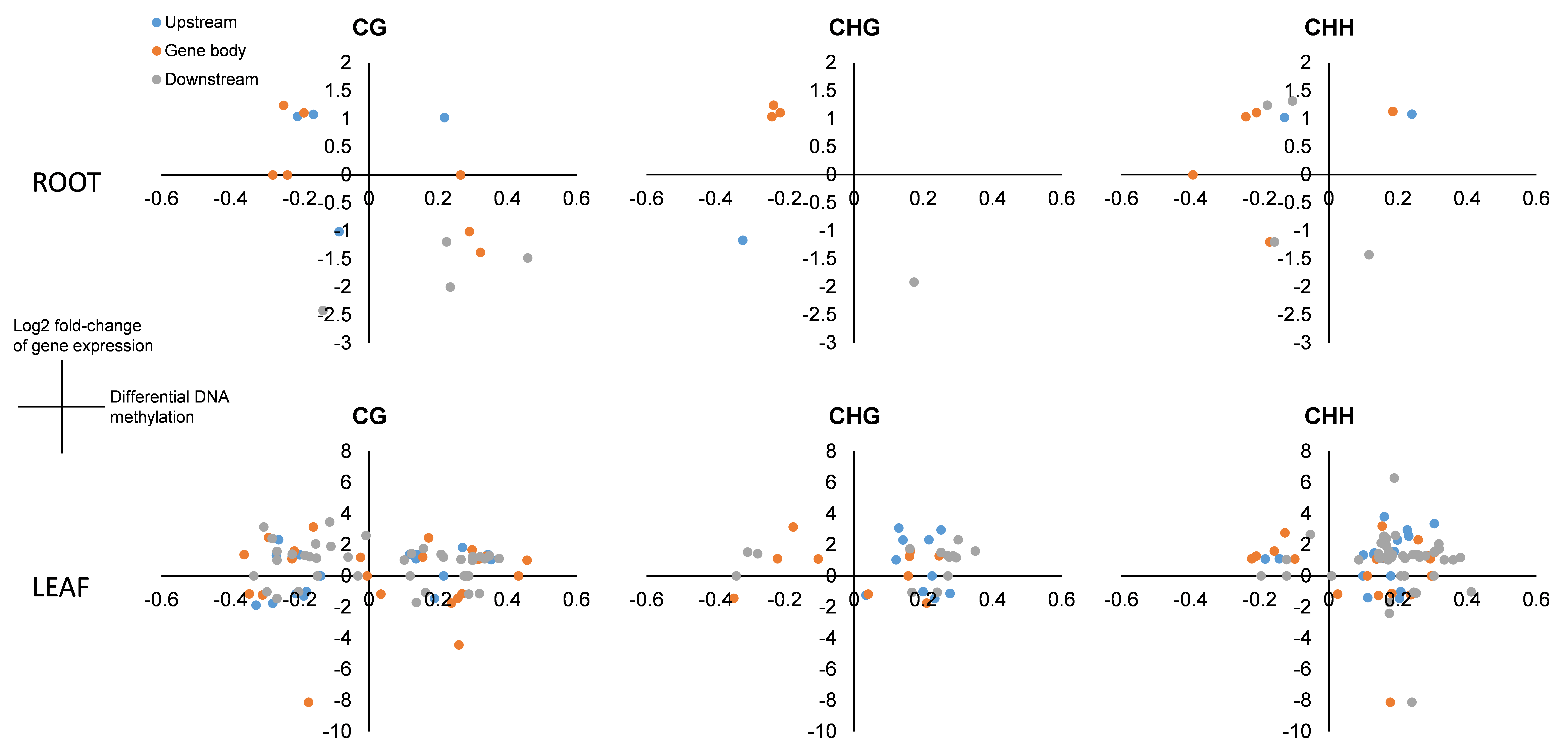

Supplement: Supplementary file 10 — Figure S4. The correlation of differential gene expression and DNA methylation changes between spaceflight and ground control in CG, CHG, and CHH contexts. The scatterplots were generated using Log2 fold-change of expression levels and average differential methylation levels in CG, CHG or CHH contexts in the location of upstream, gene body and downstream of the differentially expressed genes in roots and leaves. The data can be located in Additional file 5: Table S5. (TIF 702 kb) [file 12864_2019_5554_MOESM10_ESM.tif]
